# Learning From New State Initiatives in Financing Long-Term Services and Supports

**DOI:** 10.1093/geroni/igaa057.2530

**Published:** 2020-12-16

**Authors:** Marc Cohen, Eiileen Tell, Bonnie Albright

**Affiliations:** 1 University of Massachusetts Boston, Boston, Massachusetts, United States; 2 ET Consulting, LLC, Boston, Massachusetts, United States

## Abstract

A number of states are taking concrete action on long-term services and supports (LTSS) financing which provides an opportunity to learn about the reasons why they are doing so, to identify the coalitions that have come together in support of such actions, and to understand factors associated with the choice of particular strategies and approaches. The purpose of the presentation is to report on a comparative qualitative study across six leading-edge states—Washington State, Hawaii, Maine, Minnesota, California, and Michigan—in order to describe their activities and identify and analyze commonalities and differences in their specific approaches and programs. An overarching goal is to help state officials, consumer advocates, and interested LTSS providers understand the strategies and approaches that other states—who may be further along in their development—are taking in this area so that they might have insights into strategies that might be a fit for their state.

